# Navigation Systems for the Blind and Visually Impaired: Past Work, Challenges, and Open Problems

**DOI:** 10.3390/s19153404

**Published:** 2019-08-02

**Authors:** Santiago Real, Alvaro Araujo

**Affiliations:** B105 Electronic Systems Lab, ETSI Telecomunicación, Universidad Politécnica de Madrid Avenida Complutense 30, 28040 Madrid, Spain

**Keywords:** assisting systems, navigation systems, perception, situation awareness, visually impaired

## Abstract

Over the last decades, the development of navigation devices capable of guiding the blind through indoor and/or outdoor scenarios has remained a challenge. In this context, this paper’s objective is to provide an updated, holistic view of this research, in order to enable developers to exploit the different aspects of its multidisciplinary nature. To that end, previous solutions will be briefly described and analyzed from a historical perspective, from the first “Electronic Travel Aids” and early research on sensory substitution or indoor/outdoor positioning, to recent systems based on artificial vision. Thereafter, user-centered design fundamentals are addressed, including the main points of criticism of previous approaches. Finally, several technological achievements are highlighted as they could underpin future feasible designs. In line with this, smartphones and wearables with built-in cameras will then be indicated as potentially feasible options with which to support state-of-art computer vision solutions, thus allowing for both the positioning and monitoring of the user’s surrounding area. These functionalities could then be further boosted by means of remote resources, leading to cloud computing schemas or even remote sensing via urban infrastructure.

## 1. Introduction

Recent studies on global health estimate that 217 million people suffer from visual impairment, and 36 million from blindness [1]. The affected have their autonomy jeopardized in terms of many everyday tasks, with the emphasis being placed on those that involve moving through an unknown environment.

Generally, individuals rely primarily on vision to know their own position and direction in the environment, recognizing numerous elements in their surroundings, as well as their distribution and relative location. Those tasks are usually grouped under the categories of “orientation” or “wayfinding,” while the capability to detect and avoid nearby obstacles relates to “mobility.” A lack of vision heavily hampers the performance of such tasks, requiring a conscious effort to integrate perceptions from the remaining sensory modalities, memories, or even verbal descriptions. Past work described this as a “cognitive collage” [2].

In this regard, a navigation system’s purpose is to provide users with required and/or helpful data to get to a destination point, monitoring their position in previous modeled maps. As we will see, researchers working in this field have yet to find effective, efficient, safe, and cost-effective technical solutions for both the outdoor and indoor guidance needs of blind and visually impaired people.

Nevertheless, in recent years, we have seen unprecedented scientific and technical improvements, and new tools are now at our disposal to face this challenge. Thus, this study was undertaken to re-evaluate the perspective of navigation systems for the blind and visually impaired (BVI) in this new context, attempting to integrate key elements of what is frequently a disaggregated multidisciplinary background.

Given the purpose of this work, its content and structure differ from recent reviews on the same topic (e.g., [3,4]). Section 2 presents a historical overview that gathers together previous systems in order to present a novel survey of the principles, key points, strategies, rules, and approaches of assistive device design that are currently applicable. This is particularly important in the field of non-visual human‒machine interface, as the perceptual and cognitive processes remain the same. Next, Section 3, on related innovation fields, reviews several representative devices to introduce a set of technical resources that are yet to be fully exploited, e.g., remote processing techniques, simultaneous localization and mapping (SLAM), wearable haptic displays, etc. Finally, Section 4 and Section 5 include a brief introduction to user-centered design approaches, and a discussion of the currently available technical resources, respectively.

## 2. Background on Guidance and Navigation Systems for the Visually Impaired

This section describes general aspects of the classic design of guidance and navigation systems for the visually impaired from a historical perspective; in order to see the development and results, as well as to provide future designers with an overall view of device enhancements, which have taken place through a process of trial and error. As stated before, it is important to take into consideration that some of these classic approaches and their impact on the targeted public could even be applicable to current device design.

### 2.1. The Beginnings of Electronic Travel Aids

Over the last 70 years, researchers have worked on various prototypes of electrical obstacle detection devices for BVI people known as electronic travel aids (ETA). This was mainly caused by the fast development of radar and sonar systems, which was encouraged by the Second World War. Some of the most representative prototypes are Leslie Kay’s sonar-based Sonic Torch and Binaural Sonic Guide. Both of these will be described in Section 2.2.

The main reason why most of these first devices worked with ultrasonic signals instead of optic or radio frequency seems to lie in propagation speed [5]: the large reflection delay of sound waves allowed them to be used for distance measurements (sonar). On the other hand, systems like Laser Cane [6] resorted to techniques such as optical triangulation that resulted in less precision.

Other renowned sonar-based devices developed in the 1960s and 1970s were Russell’s PathSounder [7], the Nottingham Obstacle Detector [8] (Blind Mobility Research Unit, Nottingham University) and the Mowat Sensor [9]. All of them had similar characteristics, differing mainly in beam width and user interface, where the latter used sounds and/or vibrations to inform the user about the presence or absence of obstacles and, sometimes, even allowed them to make range estimations.

Later, in the 1980s, ETA gradually began to add processing capabilities to their designs, allowing them to further expand, filter, or make judgements about the sensors’ collected data (e.g., Sonic Pathfinder [10]). Also, user interfaces were improved by making them more efficient and user-friendly (e.g., by including recording speech [11]).

### 2.2. Sensory Substitution Devices

Sensory substitution, which derives from neuroplasticity theory, refers to the capability of the brain to assimilate information belonging to one specific sensory channel through another. Thus, it rapidly became a complementary field to the abovementioned ETA development.

**Figure 1:**
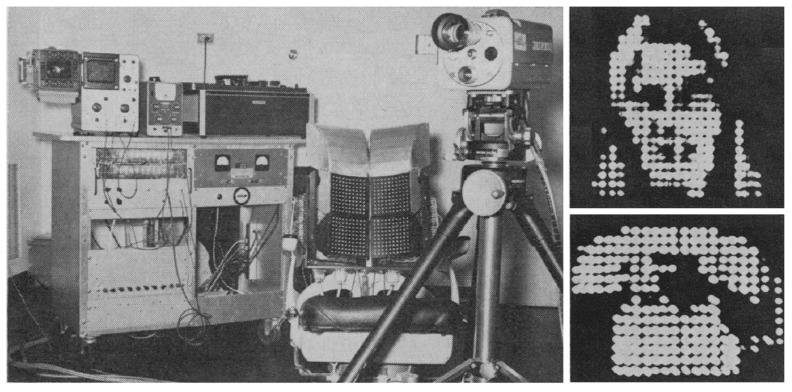
The “Tactile Television” by Paul Bach-y-Rita et al.

In this context, Paul Bach-y-Rita started collaborating with Carter Compton Collins et al. in 1964 to develop systems capable of making blind individuals able to perceive visual information through haptics [12]. Their first device projected images captured by a camera onto the skin through vibrations using a matrix of 20 × 20 haptic actuators, as displayed in Figure 1 [13]. Later surveys showed that both the blind and the blindfolded could “determine the position of visual objects, their relative size, shape, number, orientation, direction, and rate of movement,” and also “track moving targets”; even though it took a long time, the skin on the back could not handle the “vibratory-image” resolution [14] and the large amount of data observed in outdoor tests easily overloaded the user.

Despite its limitations, this project led to a number of similar systems culminating with BrainPort, a version currently available on the market [15]. This device is based on a tongue electrotactile interface: the tactile stimulus was artificially induced with surface currents in the tongue targeting each area’s corresponding afferent nerves. Some years later (2016), a study was conducted to evaluate the functional performance of BrainPort in profoundly blind individuals [16], with encouraging results from object recognition and basic orientation and mobility tasks.

Most subsequent visual‒tactile sensory substitution devices kept mapping point-for-point camera images into haptic replicas by means of mechanical elements or electrotactile interfaces (e.g., Forehead Retina System [17], HamsaTouch [18]). On the other hand, systems like the Electro-Neural Vision System (ENVS) or Haptic Radar [19], which will both be further described in later sections, focused on providing users with distance measurements from nearby obstacles so as to give them a rough, yet intuitive, notion of their surroundings.

Conversely, visual‒auditory sensory substitution has experienced far more improvements over the years. The first devices developed were Kay’s Sonic Torch [20] and Sonic Guide [21]. These devices moved the sonar reflected signal spectrum within the hearing range, leaving the task of feature recognition through sound up to the user. Some of them could even identify elements such as poles, vegetation, etc. Again, consequently, large amounts of data overloaded the user; a fact that led to solutions such as using a narrower beam width to attenuate background noise.

Later visual‒auditory designs focused on mapping images and/or proximity sensor readings into sounds in a way that could be easily deciphered by the brain. Leaving aside projects like UMS’s NAVI [22] or Hokkaido University’s mobility aid, which tried to enhance the Sonic Guide original design by replicating the echolocation of bats [23], one of the most well-known projects was Peter B. L. Meijer’s vOICe [24].

Throughout the years since its development, vOICe has been studied from different perspectives, from achieved visual acuity [25] to its potential when integrated with 3D cameras. Also, some user interviews revealed what seems to be acquired synesthesia, as they reported recovering visual perceptions such as depth [26].

Lastly, another approach exemplified by La Laguna University’s Virtual Acoustic Space [27] resorts to human hearing to recognize some 3D space characteristics from room reverberation, sound tone, etc. By means of stereo-vision 3D recording and head-related transfer function (HRTF) processed sounds, the device could reproduce virtual sound sources located over the captured surfaces through the user’s headphones. As the researchers stated: “the musical effect of hearing this stimulus could be described as perceiving a large number of raindrops striking the surface of a pane of glass.” Later tests showed how blind subjects could make use of these sounds to build a basic schematic diagram of their surroundings [27].

### 2.3. Navigation Systems for the Visually Impaired

From their birth until 1985, ETAs were not well received by the public: studies such as [5] from the USA, one of the leading countries in the research and development of devices for the BVI, stated that no more than 3000‒3500 devices were sold. Even that number does not seem accurate, as “very little is known about who purchased these ETAs.”

Those first designs focused mainly on providing obstacle avoidance support. Using them as stepping stones, numerous devices were then developed as enhanced versions in terms of weight, cost, consumption, reliability, etc. For instance, Bat K Cane [28] is a commercial sonar-based ETA designed by Leslie Kay et al. after SonicGuide. Other similar examples are UltraCane and MiniGuide [28,29], a built-in cane and hand-held device, respectively. These make use of vibrations to provide the user with adapted data from the ultrasound transductors.

Alternatively, sensory substitution device research opted for conveying visual perceptions to the BVI mainly via acoustics or haptics. Nevertheless, as mentioned earlier, the large cognitive load limited the amount of data that could be assimilated efficiently by the user, which consequently reduced the overall impact, and specifically those advances related to mobility.

Hence, neither ETAs nor general-purpose sensory substitution devices could help users reach a destination by themselves. That deficiency drove researchers to develop navigation systems that are specially adapted for the BVI, with the first known devices dating from the 1970s and 1980s.

Those devices rapidly incorporated computer-modeled maps of the environment, and required several built-in sensors and landmarks for keeping track of their position (e.g., [30]). In particular, the use of odometry became widely adopted, as can be seen in projects like Michigan University’s GuideCane [31], preceded by NavBelt [32]. These systems made use of both position and ultrasound transducer data to guide BVI users to a nearby destination while avoiding obstacles. However, with the proliferation of portable, lightweight devices, solutions that did not require continuous floor contact were preferred. Probably one of the technical advances that had the most impact in this context would be the arrival of global navigation satellite systems (GNSS), specifically the Global Positioning System (GPS), which became fully operational in April 1995.

In 1985, both C. C. Collins [12] and Jack M. Loomis [33] proposed applying GPS for BVI guidance. Later, by 1993, Loomis et al. developed their first prototype of the UCSB Personal Guidance System (UCSB PGS), a GPS-based portable device conceived as a complement to the cane that could lead the user on an outdoor route, though it did not offer any obstacle avoidance support.

The UCSB PGS project focused on designing the user interface and the geographic information system (GIS) before finally ending in 2008. Various modalities of haptic and acoustic input/outputs were tested [34], from speech interfaces to a hand-held tool made to convey descriptions of the surroundings according to which direction it was being pointed in. This solution is analogous to that of Talking Signs [35]. Among the output modalities, the researchers prioritized simulating virtual sound sources along the route, similar to what was previously seen in Virtual Acoustic Space, as it gave much better results in terms of cognitive load, time to complete the course, distance traveled, etc. [36], and it was highly rated in after-test surveys. Also, open headphones allowed the user to hear the surroundings, which mostly compensated for one of the most significant inconveniences preventing its usage in early stages.

From then onwards, numerous systems made for BVI people’s guidance relied on GNSS measurements, mostly GPS supported by dead reckoning navigation and GNSS augmentation technology. UCSB PGS is one of the first examples, reporting an accuracy of nearly 1 m by the combination of differential GPS plus inertial navigation. Some years later, Trekker, BrailleNote GPS, and other related products rapidly became available on the market. Furthermore, projects like the European Tormes and PERNASVIP [37], with the contribution of the European Space Agency, pursued enhanced GNSS positioning for BVI people guidance. For example, PERNASVIP’s technical objectives included locating “visually disabled pedestrians in urban environments within a 4-m accuracy, 95% of the time, with less than 15 s of the time to first fix.” Regretfully, mainly due to multipath errors in some urban areas, these specifications were only partially achieved.

As can be seen, locating technology became the backbone of navigation systems. Therefore, because of limited coverage by GNSS—e.g., indoor signal obstruction—and inertial navigation accumulated error, complementary systems were needed to keep track of users along their route.

Some of the preferred solutions were networks consisting of:

- Ultrasound transmitters: As an illustrative case, the University of Florida’s Drishti project [38] (2004) applied this kind of technology to BVI people guidance, combining differential GPS outdoors and an ultrasound transmitter infrastructure indoors. As for the latter, a mean error of approximately 10 cm and 22 cm maximum, was observed. However, the accuracy may be easily degraded due to signal obstruction, reflection, etc.
- Optical transmitters: By 2001, researchers from Tokyo University developed a BVI guidance system made of optical beacons, which were installed in a hospital [39]. The transmitters were positioned on the ceiling, with each one sending an identification code associated to its position. The equipment carried by the users read the code in range, then reproduced recorded messages accordingly. Another system worth mentioning belongs to the National University of Singapore [40] (~2004). This time the position was inferred by means of fluorescent lights, each of these lights having its own code to identify the illuminated area. As can be seen, this line of work has similar features to those of Li-fi.
- RFID tags: Many of the technical solutions for positioning services were based on an infrastructure of beacons, be they radio frequency, infrared, etc. However, the subsequent costs of installation and maintenance, or their rigidity against changes in the environment (e.g., furniture rearrangement), were points against their implementation. To make up for these problems, RFID tag networks were proposed. Whereas, active tag costs are usually in the tens of dollars, passive tags cost only tens of cents. Also, as batteries are discarded, the network lifetime increases while maintenance costs are lowered, thus making them attractive solutions for locating systems. Even though their range only covers a few meters, range measuring techniques based on receive signal strength (RSS), received signal phase (RSP) or time of arrival (TOA) could be applied [41]. However, the estimation of the user’s position is usually that of the tag in range. As an example of this line of work, the University of Utah launched an indoor Robotic Guide project for the visually impaired in 2003 [42]. One year later, their prototype collected positioning data from a passive RFID network with a range of 1.5 m, effectively guiding test users along a 40-m route. By 2005, their installation in shopping carts was proposed [43]. In line with this, the PERCEP’ project [44] provided acoustic guidance messages by means of a deployment of passive RFID tags and an RFID reader embedded in a glove. RFID positioning will be widely adopted in the coming years, becoming one of the classic solutions. Nevertheless, the applications are not only limited to this area. For example, they were also found suitable to search for or identify distant objects [45].

Alternatively, another possible implementation is to correlate the data collected by different sensors with a 3D map of the environment. This was exemplified by the work of Andreas Hub et al., i.e., in their subsequent hand-held [46] and built-in helmet prototypes [47].

These devices made use of techniques such as WiFi RSS measurements, inertial navigation, and stereo-vision for positioning. Furthermore, the data gathered by these sensors were applied to the recognition of previously modeled elements, e.g., pedestrians. Although error-prone, this functionality was further enhanced by delimiting the set of possible nearby elements, as some of them were associated to a static or semi-static position (e.g., table, chair, etc.).

From then on, most navigation systems for the BVI would resort to a combination of technologies, which are usually classified as indoor and/or outdoor solutions. Also, they started to gather complementary data from external sources through the net.

**Figure 2:**
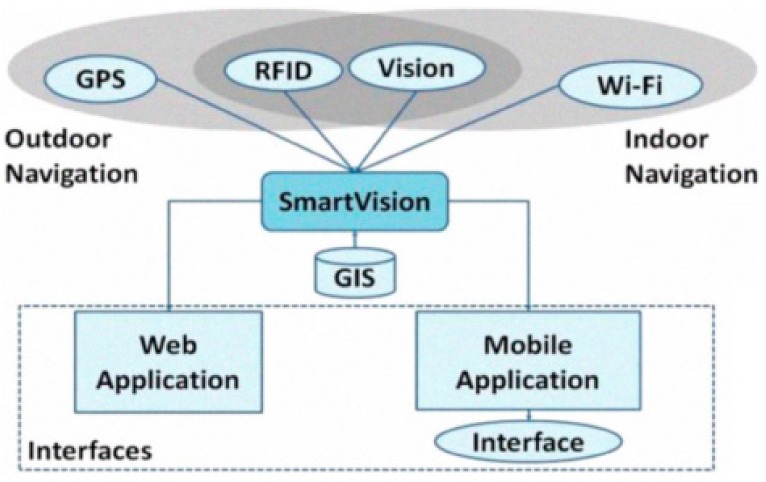
The SmartVision project: a schematic diagram.

This can be exemplified by the schematic diagram of the SmartVision project [48] shown in Figure 2. As illustrated in the previous figure, stereo vision was applied for vision positioning, and in subsequent projects included obstacle recognition functions, although it again resulted in poor performance when it came to reliability, accuracy, etc. [49]. Therefore, the locating system would effectively rely on external infrastructure (GPS, RFID, Wi-Fi). Positioning data were then combined with maps and points-of-interest (POI) available on a geographic information system (GIS) server, and thereafter offered directly to users.

From then on, various indoor positioning technologies were tested, some of which were based on Ultra-Wide Band (UWB) [50,51], passive Infrared Radiation (IR) tags [52], or Bluetooth low energy (BLE) beacons [53] combined with inertial sensors [54], and even some that exploited the magnetic signature of a building’s steel frame [55]. Among them, UWB technology stands out mainly because of its sub-meter accuracy (e.g., 15‒20 cm in [50]) and robustness to multipath interference, an issue inherent to both indoor and outdoor positioning. However, navigation through indoor scenarios usually does not require sub-meter accuracy due to similar patterns between scenarios, a reduced set of potentially hazardous elements, or a reduced size of the environment, which eases orientation and mobility tasks.

Nevertheless, as navigation systems continued their development, and the amount of information collected for blind navigation grew larger, the need for efficient user interfaces became even more apparent.

Several classic solutions involved speech, beginning with recorded messages (e.g., Guide Dog Robot, Sonic Pathfinder); later, speech synthesis and recognition were also gradually incorporated (e.g., Tyflos [56]). At this point, sensory substitution became an attractive solution for blind navigation system user interfaces, more so when the user needed the system to rapidly provide detailed information regarding its immediate surroundings, while maintaining a low cognitive load.

**Figure 3:**
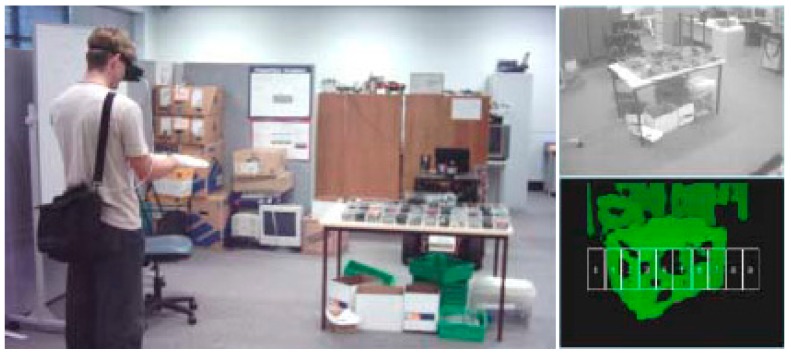
The ENVS project.

In line with this, the ENVS project [57] is another representative example that conveys depth perceptions through haptics. Again, it makes use of a pair of cameras to capture the 3D environment and present it to the user as tactile stimuli in their fingers. Distance data were encoded in the pulse width of electrotactile stimulation signals. If the gloves were aligned with the cameras, it seemed as if things were being touched at a distance. Furthermore, the tests showed how this solution allowed users to intuitively assimilate information from 10 virtual proximity sensors (Figure 3) with a relatively low cognitive load.

By 2005, the device incorporated a built-in GPS and compass to allow for outdoor guidance [58]. Orientation data were passed on to the user through the electrotactile gloves, overlapping the distance-encoding signals.

## 3. Related Innovation Fields

This section focuses on related R&D technological areas that currently benefit from greater attention and investment, as they could constitute some of the most important contributors in order to achieve BVI mobility self-sufficiency.

### 3.1. Mixed Reality

In recent years, virtual and real environments have been slowly breaking down barriers and becoming closer, e.g., by virtualizing physical objects or an individual’s movement, mixing virtual and real elements in an immersive scenario, etc.

When forming a picture of mixed reality, system latencies ranging between tenths or even hundredths of seconds are often required. Specifically, complying with that limitation when virtualizing features of real elements led to the development of low-latency techniques and commercial products for recording the three-dimensional environment.

Such circumstances would boost the implementation of functionalities needed for navigation systems such as obstacle detection and recognition. This would then be exemplified by projects like NAVI [59], based on Microsoft Kinect.

Soon enough, the high potential of applying computer vision for positioning was further exploited. Simultaneous locating and mapping technology (SLAM), which can be found in Google’s Project Tango, allowed for centimeter-level accuracy indoor positioning. Project Tango and related technologies such as Intel RealSense provided vision positioning solutions, with reported cases of application in commercially available drones like Yuneec’s Typhoon H. Specifically, the applications for BVI navigation that had been previously contemplated materialized in the development of various prototypes. For example, the Smart Cane system [60] used a depth camera and a server for SLAM processing that allowed for six degrees-of-freedom indoor location, plus obstacle detection features. Also, ISANA [61] exploited Project Tango for indoor wayfinding and obstacle detection, using compatible hardware platforms (i.e., Phab 2 or Yellowstone mobile devices) and haptic actuators embedded in a cane. Analogously, in [62] a novel prototype is described that used Tango and Unity, a game engine, to capture the user’s movement in a continuously updated virtual replica of the indoor environment. In addition to wayfinding and mobility assistance, SLAM techniques were also used for tasks such as face recognition [63].

**Figure 4:**
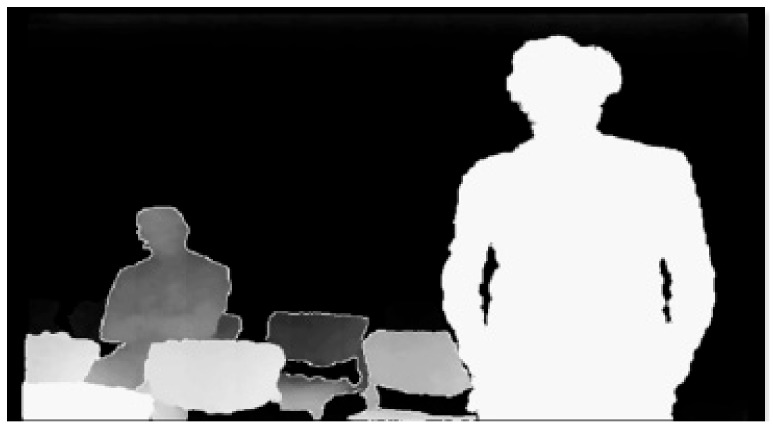
A VA-ST Smart Specs captured image.

Another remarkable application of this technology for VI people’s guidance lies in the user interface. One solution proposed by Stephen L. Hick et al., from Oxford University, exploited the residual vision by enhancing 3D perceptions with simplified images emphasizing depth (Figure 4) [64]. They recently tried to access the market with their Smart Specs [65] glasses, with VA-ST start-up funding.

Alternatively, mixed reality allows users to interact with virtual elements overlapping with their actual surroundings, thus providing intuitive cues of orientation, distance from and shapes of objects, etc.

The usage of virtual sound sources to guide pedestrians along a route is one of the classic solutions seen in projects like UCSB PGS, or even Haptic Radar. The latter combined its original IR-based obstacle avoidance system with virtual sound guidance, which resulted in positive after-test appraisals [66]. Nevertheless, some criticisms and suggestions were made, mainly in relation to the area covered by the IR sensors and the vibrational interface.

Also, virtual sounds could not only be applied for guidance, but also for at least several tasks that involved 3D enhanced perception, as previously seen in Virtual Acoustic Space.

**Figure 5:**
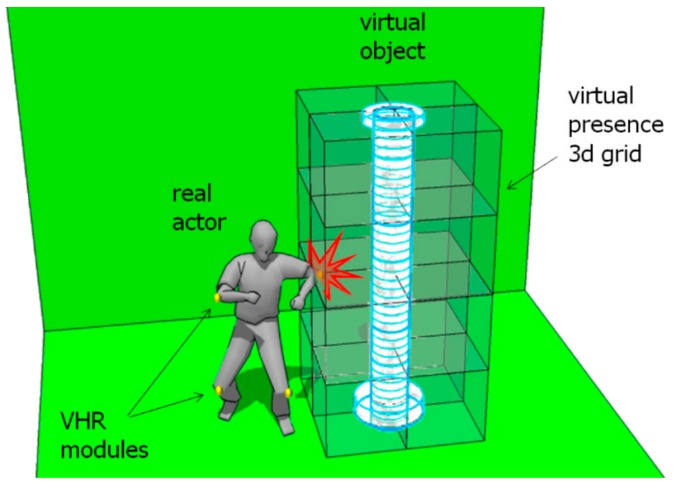
Virtual Haptic Radar project.

Aside from solutions based on sound, virtual tactile elements were also studied, albeit apparently less. The Virtual Haptic Radar project [67], originating from Haptic Radar, is a representative example. It substituted its predecessor’s IR sensors by the combination of a three-dimensional model of the surroundings plus an ultrasonic-based motion capture system worn by the user. As described in Figure 5, once the user reached a certain area near the object, warning vibrations were triggered accordingly.

However, one of the main problems hampering tactile-based solutions is the haptic interfaces available. Most portable designs seem to resort to mechanical components, thus causing a conflict between their bulkiness and the subtlety of the induced perceptions. Alternatives such as electrotactile devices remain experimental so far.

### 3.2. Smartphones

Over the last decade smartphones, among other portable devices, have gradually included a variety of features that would make them resourceful platforms for developers, some of which will be discussed next.

As a stand-alone device, a smartphone shows a high and rapidly increasing processing capacity in comparison with its price. Additionally, it incorporates a diverse set of built-in tools and sensors, like cameras, GNSS modules, accelerometers, gyroscopes, or NFC readers. In addition, close-range communication via Bluetooth or Wi-Fi further expands the previous assortment of uses, e.g., by means of external sensors for obstacle detection, high-precision RTK-GNSS modules, etc.

On the other hand, mobile networks keep on improving with each new release, leading to the usage of remote resources. In accordance with this, cloud computing services are nowadays commercialized at various levels of abstraction, such as infrastructure (IaaS), platforms (PaaS), or software (SaaS). Remarkable examples in our line of work, as will later be shown, are artificial vision SaaS, as offered by Google or Microsoft, providing developers with APIs to get access to Google Cloud Platform and Microsoft Cognitive Services resources, respectively.

An additional aspect to be aware of is the acceptance of smartphones specifically by BVI users [68]. Even before accessibility for handicapped people made its way into software design standards, as can be seen in Apple’s iOS, mobile phones have progressively become widely adopted for calls or to send text messages. Now, with the generational change, the number of users of these new technologies has further increased.

**Figure 6:**
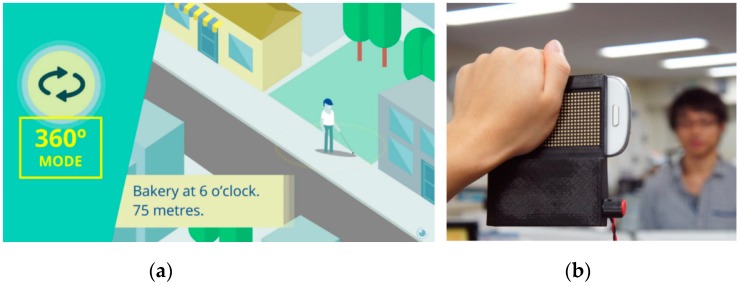
Lazzus (**a**). HamsaTouch (**b**).

In this environment, research on navigation systems for BVI users found a new field to exploit, e.g., the BLE-based NavCog smartphone application [53] or purely inertial prototypes [69] for indoor wayfinding. Regarding general-purpose sensory substitution, a few visual‒auditory systems soon became publicly available software applications, e.g., EyeMusic [70,71], or even the classic vOICe [72]. Conversely, visual‒tactile sensory substitution systems were once again comparatively scarce. One example would be HamsaTouch, seen in Section 2.2, which recreates Bach-y-Rita’s and Collins et al.’s prototypes in a smartphone equipped with a haptic electrotactile display (Figure 6b). On the other hand, applications such as Seeing AI [73] or TapTapSee [74] provide users with verbal descriptions of captured images, making use of remote processing resources in a cloud computing schema.

Nevertheless, the focus of attention was placed on GNSS-based outdoor navigation. Next, some representative examples of available applications are briefly described:

- Moovit [75]: a free, effective, and easy-to-use tool that offers guidance on the public transport network, managing schedules, notifications, and even warnings in real time. It is one of the assets for mobility tasks recommended by ONCE (National Organization of Spanish Blind People).
- BlindSquare [76]: specifically designed for the BVI, this application conveys the relative location of previously recorded POIs through speech. It makes use of Foursquare’s and OpenStreetMap’s databases.
- Lazzus [77]: a paid application, again designed for BVI users, which coordinates GPS and built-in motion capture and orientation sensors to provide users with intuitive cues about the location of diverse POIs in the surrounding area, even including zebra crossings. It offers two modes of operation: the 360° mode verbally informs of the distance and orientation to nearby POIs, whereas the beam mode describes any POI in a virtual field of view in front of the smartphone. Its main sources of data are Google Places and OpenStreetMap.

Some of these functionalities are also shared by an increasing number of commercially available applications, each with specific characteristics and improvements. For example, Seeing AI GPS [78] includes solutions analogous to 360° and beam modes of Lazzus plus pre-journey information; NearBy Explorer offers several POI notification filters, etc.

### 3.3. Wearables

So far, bone conduction headphones and smart glasses with a built-in camera have mainly been used for BVI mobility support. Furthermore, as the size and cost of sensors and microprocessors further decreased, and given the advantages of wearable devices, the development of designs specifically aimed at these people has been slowly boosted.

**Figure 7:**
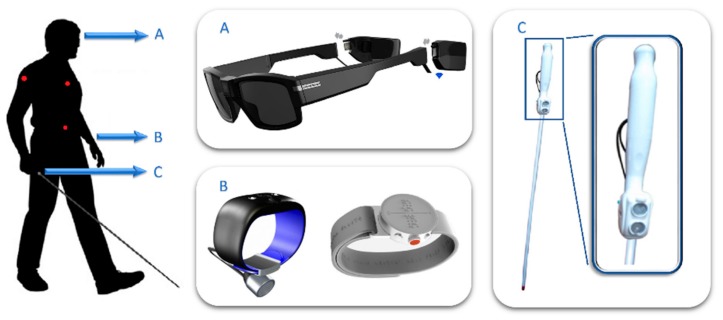
Wearables for the BVI: common placements.

Some of the main points in favor of wearable designs include the sensors’ wider field-of-view, the usage of immersive user interfaces, or users’ request for discreet, hands-free solutions. In Figure 7, some strategic placements of these sensors and interfaces are shown, including a few examples of market-available products.

Firstly, regarding the sensors’ field-of-view, some devices rely on the user to scan their surroundings, whereas others resort to intermediary systems that monitor the scene. Among them, the first strategy was therefore to look for placements that eased “scanning movements,” placing sensors on the wrist (Figure 7B), the head (Figure 7A) or embedded in the cane (Figure 7C). Specifically, systems corresponding with Figure 7B,C tended to imitate the features of the first ETA. This was exemplified by Ultracane, SmartCane (Figure 7C) or Sunu-band [79] (Figure 7B), as all of them offered obstacle detection functionalities supported by ultrasound proximity sensors via a vibrational user interface. On the other hand, the third category of wearables (Figure 7A) was usually seen in camera-based sensory substitution or artificial vision systems, e.g., Seeing AI, Orcam MyEye [80], BrainPort, or even vOICe.

Conversely, the second strategy generally opts for a wider field-of-view, thus sensors were often positioned in relatively static and non-occlusive placements all over the torso (red dots in Figure 7). That was the case with Toyota’s Project Blaid [81], a camera-based, inverted-U-shaped wearable that rested on the user’s shoulders. Among its functionalities, it pursued object and face recognition, with an emphasis placed on elements related to mobility such as stairs, signals, etc.

Regarding user interfaces, speech and Braille made up the first solutions for acoustic and tactile verbal interfaces, coupled with headphones and braille displays. As an example, Figure 7B shows the “Dot” braille smartwatch.

Other kinds of solutions strived for a reduced cognitive load by means of intuitive guidance cues, usually exploiting the innate space perception capabilities of touch and hearing. Many examples have been mentioned in this text, from Virtual Acoustic Space or UCSB PGS to Haptic Radar. Non-occlusive headphones and vibratory interfaces are some of the devices most commonly used as they benefit from a low cost, a reduced-weight design, etc., while still being able to generate immersive perceptions such as virtual sound sources, or the approach to tactile virtual objects, as seen initially in Haptic Radar, and later in Virtual Haptic Radar.

This latter approach is also found in the Spatial Awareness project, based on Intel RealSense. The developed prototype conveys distance measurements through the vibration of eight haptic actuators distributed over the user’s torso and legs.

## 4. Challenges in User-Centered System Design

As will be discussed, a major flaw in the design of navigation systems for BVI users seems to lie in a set of reiterated deficiencies concerning the knowledge of the users’ needs, capabilities, limitations, etc., despite the great amount of work that has accumulated over the last few decades. Thus, this section will attempt to gather key user-centered design features prior to a further discussion of system design in Section 5.

One of the first problems faced in the development of assistive technology is the heterogeneity of the targeted public [82]. The assistance required is related to the users’ residual vision, among other circumstances, such as physical or sensory disabilities deriving from the ageing process that should be noted (81% of the BVI are aged above 49 years [1]). In particular, this section will focus on blindness as the most severe case of disability, so as to provide the reader with enough data to infer the needs of specific users.

Several user requirements concerning navigation systems for the blind have often been addressed. Firstly, regarding the disposal of environmental information, some typical features to offer are [5]:

1. “The presence, location, and preferably the nature of obstacles immediately ahead of the traveller.” This relates to obstacle avoidance support.
2. Data on the “path or surface on which the traveller is walking, such as texture, gradient, upcoming steps,” etc.
3. “The position and nature of objects to the sides of the travel path,” i.e., hedges, fences, doorways, etc.
4. Information that helps users to “maintain a straight course, notably the presence of some type of aiming point in the distance,” e.g., distant traffic sounds.
5. “Landmark location and identification,” including those previously seen, particularly in (3).
6. Information that “allows the traveller to build up a mental map, image, or schema for the chosen route to be followed.” This point involves the study of what is frequently termed “cognitive mapping” in blind individuals [83].

Whilst the first ETAs were oriented to the first category of information, solutions that placed virtual sound sources over POIs easily covered points (4) and (5), and solutions based on artificial vision could provide data in any category.

One key factor to be aware of in this context is the theory behind the development of sensory substitution devices, which has been mentioned throughout the text when describing the “cognitive load” or “intuitiveness” of some user interfaces. At this point, the work in [84] is highlighted as it introduces the basics.

In the first place, some major constraints to be considered are the difference of throughput data capability between sensory modalities (bandwidth), and the compatibility with higher-nature cognitive processes [84]. Two respective examples of these constraints would be the overloading of touch seen in numerous attempts to convey visual perceptions [85], and the inability to decipher visual representations of sounds, even though vision has comparatively more ‘bandwidth’ than hearing.

Some other main factors would be the roles of synesthesia and neuroplasticity, or even how intelligent algorithms can be used to filter the information needed in particular scenarios [84].

Once it was proven that distant elements can be recognized through perceptions induced by sensory substitution devices of vision (Section 2.2), thus straying into the field of “distal attribution” (e.g., [84,85]), it started an ambitious pursue of general-purpose visual‒tactile and visual‒auditory devices. Several recent studies in neuroscience showed the high potential of this field [86,87], as areas of the brain though to be associated to visual-type tasks, e.g., involved in shape recognition, showed activity with visual-encoded auditory stimulation.

Nevertheless, given the limitations of the remaining senses to collect visual-type information, it is usually necessary to focus on what users require to carry out specific tasks [88,89].

Lastly, the poor acceptance of past designs by their intended public should be taken into account; a recent discussion on this topic can be found in [88]. In line with this, an aspect that was recently taken advantage of is the growing penetration of technology in the daily routines of BVI people, with an emphasis placed on the usage of smartphones.

**Figure 8:**
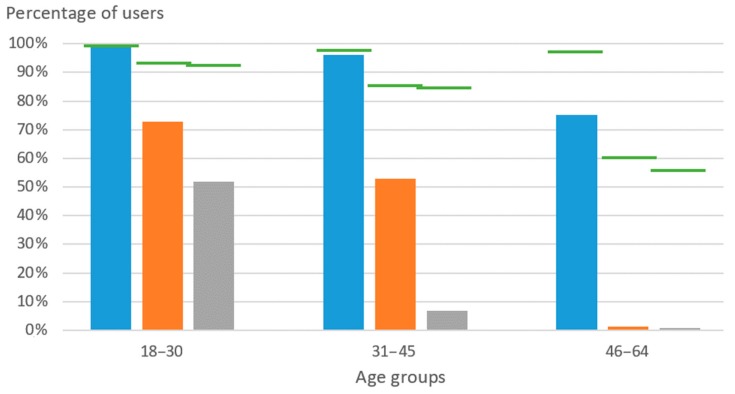
Percentages of Spanish BVI users of mobile phones (blue) and computers (orange); percentage of those who access the Internet (gray), and references to the overall population (green). Data obtained from INE and [51] (2013).

Figure 8 shows the increasing growth of mobile phone and computer use, including how many BVI people use these devices to access the Internet, a tendency likely to continue among younger generations. This trend is also reflected in the creation of entities such as Amovil, which promotes the accessibility of these devices to the BVI people, or the smartphone-compatible infrastructure of London’s WayFindr [90] (similar to [91,92]), Bucharest’s Smart Public Transport [93], or Barcelona’s NaviLens [93], which are oriented to boosting the autonomy of BVI individuals when using public transportation. In line with this, Carnegie Mellon University’s NavCog, based on a BLE network, recently added Pittsburgh International Airport to the list of supported locations [94].

## 5. Availability of Technical Solutions

Finally, this last section will delve into some general aspects of potential architectures. Functional requirements and their feasibility will be discussed according to past experiences, the available technology, and user-related needs and constraints.

**Figure 9:**
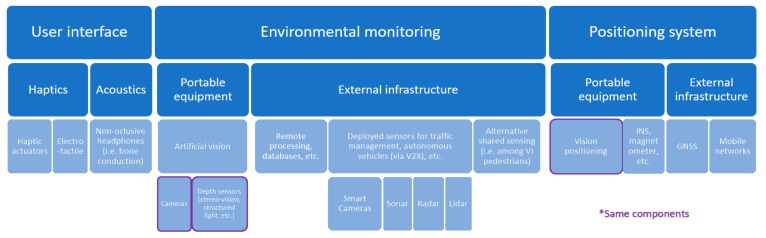
Architecture proposal for navigation assistance devices (examples included).

The discussion on this topic will be addressed according to three main functionalities of navigation systems for the blind, namely positioning systems, environment monitoring and user interface (Figure 9). The system coordinates the abovementioned modules with complementary data, such as POIs (e.g., OpenStreetMap), maps, public transportation schedules, etc. which are available via the web.

### 5.1. Positioning Systems

Focusing on assistance along a route, a navigation system needs positioning data, but its specifications may differ according to the solution pursued. For example, applications like Lazzus efficiently indicate the location and nature of POIs with accuracies of about 1 m. On the other hand, projects that simulate virtual sound sources, such as Virtual Acoustic Space, usually need cm accuracy positions, in addition to split-second time responses to match the HRTF output sounds with head movements. These are typical constraints of current mixed reality applications.

Additionally, the design of navigation systems varies depending on whether it is oriented to indoor or outdoor environments (see Section 4). This particularly affects positioning techniques, which can be further classified into portable equipment, e.g., related to dead reckoning navigation solutions, or external infrastructure that ranges from BLE beacons to GNSS. The technologies to be applied would then be chosen according to the requirements of the targeted tasks, costs, etc.

Some of the most attractive solutions are those that take advantage of already deployed infrastructure, which is reflected in the absolute prevalence of GNSS for outdoor location. It could also be combined with mobile networks, or portable alternatives such as INS and/or the previously discussed vision positioning. On the other hand, most of the indoor positioning techniques encountered, including those currently available on the market, require a beacon infrastructure deployment that easily pushes up costs, whereas usage would be extremely low.

At this point, portable devices for vision positioning show high promise for low-cost positioning, both in outdoor and indoor environments (Section 2.2 and Section 3.1). Additionally, vision-based solutions could provide data on the users’ surroundings (Section 2.2, Section 2.3, Section 3 and Section 5.2), and also play an important role in the design of sensory substitution devices (Section 2.2, Section 2.3, Section 3.1, Section 3.2, Section 4 and Section 5.3).

Whilst most GNSS and/or mobile networks can delimit user location within a few meters even in indoor scenarios (e.g., 5G [95]), vision positioning further improves it to cm precision. Furthermore, the same obstacles that degrade GNSS signals, e.g., buildings or bridges, could make fine reference points for solutions based on image processing, making up for the accumulated error characteristic of dead reckoning techniques. Some current drones, like DJI’s Phantom 4, stabilize their movements through precise location feedback based on this kind of strategy.

### 5.2. Environmental Monitoring

As seen in Section 4, navigation systems for BVI users need to gather specific data of the environment for an efficient and safe guidance.

In this context, a first distinction to make is whether any object, feature, etc., in range is fixed to a specific location, hereinafter referred to as static (e.g., stairways) or dynamic (e.g., pedestrians) elements.

Static elements could be relatively easy to handle through records of their distribution and relevant features in shared databases. This would be exemplified by Wayfindr, as the users’ closeness to BLE beacons triggers guidance cues and notifications of nearby elements. Dynamic elements, on the other hand, are to be managed with sensors such as cameras, sonar, LiDAR, etc., be they remote installations or equipment carried by the user.

As for what technology should be used to capture those dynamic elements, it depends on the specific application. Classic examples are Ultracane and Miniguide sonar-based obstacle detection devices, or those that are vision or infrared-based, described in Section 3.

Nevertheless, these mobility aids usually face strict constraints of reliability and robustness, as they could put users in potentially hazardous situations. Following this statement, three alternatives will be discussed.

Firstly, in opposition to autonomous devices, these aids can make use of the users’ judgement. Starting from the premise that raw measurement data of sensors do contain what is needed, e.g., to detect and avoid an obstacle, the issue lies in whether the user could effectively and efficiently analyze that flow of information. This delves into the domain of sensory substitution and augmentation, with Virtual Haptic Radar as an example of the potential of extended touch [96] in a context of mixed reality.

Secondly, not all orientation and mobility tasks require such extreme reliability. Common useful features could be signal detection and recognition, the detection of nearby pedestrians, etc., most of which are currently implemented in artificial vision technology. These solutions include precedent systems going back to Tyflos, the recent and market-available Seeing AI and Orcam MyEye, or current prototypes such as [97]. Also, the potential of vision-based systems would be even higher in urban areas, as they are built placing great care on what elements are visible.

Third and finally, the reliability and robustness of mobility-related tasks can be inherited from external resources, e.g., by leaning on urban monitoring infrastructures, as seen in Siemens’ InMobs project.

### 5.3. User Interface

Once the relevant data for navigation are gathered, they are then passed onto the user. However, this is one of the critical aspects in the design of products for BVI people, and usually acts as a bottleneck of the information available in numerous navigation systems.

Speech interfaces can be applicable for several tasks, e.g., when providing brief descriptions of the user surroundings, OCR, etc., as seen in Seeing AI. However, its use involves several constraints and problems. Firstly, it could mean that the user may not hear or pay attention to the environment. Simple, short messages are typically preferred, thus limiting the data provided. Secondly, the data gathered must be analyzed and filtered according to the users’ requirements at each time and place, a challenge similar to those of autonomous vehicles or drones. Thirdly, spatial cues are often non-optimal, even in the case of simple left/right indications [34]. Most of these problems could be extended to other linguistic interfaces (e.g., braille displays).

As for non-linguistic interfaces, the first limitation would be the extremely low data throughput of hearing and touch in comparison with vision, followed by the need to match the data output with “higher-nature cognitive processes” [84] (Section 4). Therefore, according to Giudice, Loomis, Klatzky et al., developers should focus on helping users to perform specific and actually needed tasks, minimizing the conveyed information, while taking advantage of the “perceptual and cognitive factors associated with non-visual information processing” [84,88].

These last factors can be exemplified by the natural cross-modal associations observed in the project vOICe, such as volume-to-brightness and pitch-to-spatial height (see “weak synesthesia” in [98]). This was even evident in Disney-supported research on color‒vibration correspondences [99], which came from the pursuit of more immersive experiences. Other illustrative cases include individuals exploiting the spatial-rich information of sound to extreme levels, e.g., the echolocation techniques shown by Daniel Kish. These techniques might be reminiscent of the first ETA described in Section 1.

Another remarkable aspect to point out is the effect on distal attribution of the correspondence between body movement and perceptions [100]. For example, in Bach-y-Rita’s et al. visual‒tactile experiments, it was observed that users needed to manipulate the camera themselves to notice the “contingencies between motor activity and the resulting changes in tactile stimulation” [84].

The use of these proprioception correspondences might be a fundamental element in the design of future orientation and mobility aids, given the good performance of past projects.

Several of the mentioned projects incorporate mixed-reality-type user interfaces, such as the virtual sound sources seen in UCBS PGS and Virtual Acoustic Space, or the virtual tactile objects of Virtual Haptic Radar. Another system worth highlighting is Lazzus, which tracks the smartphone’s position and orientation to trigger verbal descriptions according to which direction it is being pointed in. As seen with Talking Signs, these approaches have users’ support [101].

Nevertheless, some of these solutions are also affected by technical limitations. While bone-conduction earphones and head motion tracking techniques are sufficient for most sound-based applications, portable haptic interfaces are heavily constrained. Even though haptic displays such as those commercialized by Blitab could promote tactile-map approaches, portable alternatives are limited to vibrational interfaces. These devices by no means exploit the full capabilities of touch, thus hampering further exploration in fields such as the application of extended touch [96] in a context of mixed reality. However, recent advances might boost the growth of a versatile classic solution known as “electrotactile.”

This technology, which benefits from low cost, low power consumption, and lightweight design, encompasses a wide range of virtual perceptions. Nevertheless, it has an insufficient theoretical foundation in terms of neural stimulation, and several designs have revealed problems related to poor electrical contact through the skin. This could be partially compensated for by choosing placements with more adequate electrical conditions, such as the tongue (BrainPort), or by the use of a hydrogel for better control of the flow of the electrical current (e.g., Forehead Retina System), etc.

Nowadays the same BrainPort makes a market-available device that shows the feasibility of this haptic technology for some applications. In addition, over the years, subsequent prototypes have strived for various improvements, such as combining electrotactile technology with mechanical stimuli [102,103], stabilizing the transcutaneous electrode‒neuron electrical contact, albeit with closed-loop designs [104], or micro-needle interfaces [105,106], etc. Furthermore, the neural stimulation theoretical basis continues to advance through research in related fields, e.g., when developing myoelectric prostheses that provide a sense of touch via the electrical stimulation of afferent nerves.

## 6. Conclusions

Numerous devices have been developed to guide and assist BVI individuals along indoor/outdoor routes. However, they have not completely met the technical requirements and user needs.

Most such unmet aspects are currently being answered separately in several research fields, ranging from indoor positioning, computation offloading, or distributed sensing, to the analysis of spatial-related perceptual and cognitive processes of BVI people. On the other hand, smartphones and similar tools are rapidly making their way into their daily routines. In this context, old and novel solutions have become feasible, some of which are currently available in the market as smartphone applications or portable devices.

In line with this, the present article attempts to provide a holistic, multidisciplinary view of the research on navigation systems for this population. The feasibility of classic and new designs is then briefly discussed according to a new architecture scheme proposal.
